# Urinary N-telopeptide: The New Diagnostic Test for Osteoporosis

**DOI:** 10.1055/s-0038-1677483

**Published:** 2019-01-08

**Authors:** Ganesan Ram Ganesan, Phagal Varthi Vijayaraghavan

**Affiliations:** 1 Department of Orthopaedics, Sri Ramachandra University, Chennai, Tamil Nadu, India

**Keywords:** osteoporosis, DXA scan, osteopenia, telopeptide, fracture

## Abstract

**Context** Osteoporosis is a silent disease until it is complicated by trivial fall fractures. There is an increasing interest within the orthopaedic community in the noninvasive cost-effective measurement of the bone mineral density.

**Aims** The aim of the study is to assess whether urinary N-telopeptide level can be a new diagnostic tool in diagnosing osteoporosis.

**Methods and Material** This prospective study was done at Sri Ramachandra Medical Centre (SRMC) hospital from October 2015 to October 2017. The study was conducted among patients who comes to SRMC as inpatient or outpatient with suspected osteoporosis and underwent dual-energy X-ray absorptiometry (DXA) scan and urinary N-telopeptide. The inclusion criteria were women aged 65 or older, women aged less than 65 with risk factors, younger postmenopausal women with one or more risk factors, men aged 70 or older, men less than 70 with risk factors, and any above group patients who comes within 24 hours following trivial fall fractures. The exclusion criteria were pathological fracture, history of any illness affecting bone metabolism. The results from DXA scan were taken as gold standard against urinary N-telopeptide. Then the patients were divided into two groups control and study. The control group contains patients who had normal DXA, while study group contains patients having either osteopenia or osteoporosis. Based on our inclusion and exclusion criteria, 110 persons were included in the study. We had 60 study and 50 controls patients. We had 88 females and 22 males. The results obtained were statistically analyzed.

**Statistical Analysis Used** The collected data were analyzed with IBM SPSS statistics software 23.0 version. To describe about the data descriptive statistics frequency analysis, percentage analysis was used for categorical variables and the mean and standard deviation were used for continuous variables. To find the significant difference between the bivariate samples in independent groups, the unpaired sample *t* -test was used. To find the significance in categorical data, chi-square test was used. In both the earlier statistical tools, the probability value of 0.05 is considered as significant level.

**Results** In our study, we had 18.2% osteopenic and 36.4% osteoporotic patients. The mean value of urinary N-telopeptide in control was 49.8 and in case was 182.5. The standard deviation of urinary N-telopeptide value in case was 159.9.

**Conclusion** Urinary N-telopeptide can give reproducible results and be able to assist in the evaluation of the quantity as well as the quality and be a good judge of someone's risk of fracture. Hence, urinary N-telopeptide can be used as a diagnostic tool for diagnosing osteoporosis.

Osteoporosis is a silent disease until it is complicated by trivial fall fractures. These fractures causes enormous financial, medical, and personal burden to the patients and to the nation. Osteoporosis not only affects woman but affects men also. Osteoporosis in men is underrecognized and thus undertreated. 1 Till now, the gold standard for diagnosing osteoporosis is dual-energy X-ray absorptiometry (DXA) scan. DXA is a costly method to assess bone mineral density. Present scenario needs a simpler and more cost-effective method for diagnosing osteoporosis. There is an increasing interest within the orthopaedic community in the noninvasive cost-effective measurement of the bone mineral density. The aim of the study is to assess whether urinary N-telopeptide level can be a new diagnostic tool in diagnosing osteoporosis.

## Subjects and Methods

This prospective study was done at Sri Ramachandra Medical Centre (SRMC) hospital from October 2015 to October 2017. The study was conducted among patients who comes to SRMC as inpatient or outpatient with suspected osteoporosis and underwent DXA scan and urinary N-telopeptide. The sampling method in this study is probability sampling. Ethics committee approval was obtained from SRMC institutional ethics committee. The inclusion criteria were women aged 65 or older, women aged less than 65 with risk factors, younger postmenopausal women with one or more risk factors, men aged 70 or older, men less than 70 with risk factors, and any earlier group patients who comes within 24 hours following trivial fall fractures. 2 3 4 The exclusion criteria were pathological fracture, history of any illness affecting bone metabolism such as renal failure, hepatic failure, active malignancy, thyroid abnormalities, and drugs that affect bone metabolism such as steroids, anticonvulsants etc. The patients who meet the inclusion criteria were enrolled into the study. Prior informed consent was obtained from all patients.

DXA scan was done using GE healthcare Prodigy pro-DXA machine. DXA scan for right hip was done for all patients. If the patient is having fracture or operation done in the right hip, DXA scan was done in left hip or spine. In cases were DXA scan was done for hip and spine and any one area reported as normal and other as osteoporosis/osteopenia, then the patient was excluded from the study. Twenty-four hours urine was collected in a sterile plastic container. The collected specimen was sent to the centralized laboratory in SRMC. Urine sample was analyzed by enzyme-linked immunosorbent assay (ELISA) technique using Osteomark kit. The inter- and intra-assay coefficients of variation of Osteomark urinary N-telopeptide kit are 4.0 and 7.6%, respectively.

The results from DXA scan were taken as gold standard against urinary N-telopeptide. 5 6 Then the patients were divided into two groups control and study. The control group contains patients who had normal DXA, while study group contains patients having either osteopenia or osteoporosis. Routine bone profile investigations such as serum calcium, phosphorus, alkaline phosphatase, serum albumin was done for all patients.

Based on our inclusion and exclusion criteria, 110 persons were included in the study. We had 60 study and 50 controls patients. We had 88 females and 22 males. The results obtained were statistically analyzed. The collected data were analyzed with IBM SPSS statistics software 23.0 version. To describe about the data descriptive statistics frequency analysis, percentage analysis was used for categorical variables, and the mean and standard deviation were used for continuous variables. To find the significant difference between the bivariate samples in independent groups, the unpaired sample *t* -test was used. To find the significance in categorical data, chi-square test was used. In both the earlier statistical tools, the probability value of 0.05 is considered as significant level.

## Results

In our study, we had 18.2% osteopenic and 36.4% osteoporotic patients as evident from Table 1 . The mean value of urinary N-telopeptide in control was 49.8 and in case was 182.5. The standard deviation of urinary N-telopeptide value in case was 159.9 from Table 2 . From Table 3 , the independent sample test for urinary N-telopeptide clearly shows significance association with osteoporosis/osteopenia. We had totally 53 patients associated with fracture out of which 47 were cases and 6 were controls.

**Table 1:** Results of DXA scan

DXA scan	Study	Control	Percentage (%)
Normal	0	50	45.5
Osteopenia	20	0	18.2
Osteoporosis	40	0	36.4

Abbreviation: DXA, dual-energy X-ray absorptiometry.

**Table 2:** Mean values of urinary N-telopeptide

Groups	*N*	Mean	Standard deviation	Standard error mean
Study	60	182.540	159.968	20.6518
Control	50	49.839	31.3343	4.4313

**Table 3:** Independent sample test for NTx

NTx value	Levene's test for equality of variances		t-test for equality of means						
	*F*	Significance	*T*	*df*	Significance (two tailed)	Mean difference	Standard error difference	95% Confidence interval of the difference	
								Lower	Upper
Equal variance assumed	29.076	0.000	5.770	108	0.000	132.7006	22.9983	87.1140	178.2872
Equal variances not assumed			6.283	64.394	.0005	132.7006	21.1219	90.5097	174.8915

## Discussion

DXA scan will give us the quantity of bone; however, it does not give evaluation of patients bone quality. The factors that may alter or change the DXA scan results are artifacts, anatomy, machinery, location, varying technicians, and positioning of patients. Wherever there is radiation, there is always a chance of danger related to that. Any accidental exposure of radiation can cause drastic implications. However, if this is carefully done, the advantages of DXA outweigh its risk. Moreover, maximum permissible limit of precision error of technician doing DXA is around 1.7% for lumbar spine and 1.3% for femoral neck. 7 A major disadvantage of DXA is that currently, there is a lack of standardization in bone and soft tissue measurements. 8 DXA scan reports tend to vary based on hydration status of patients, tissue thickness, and soft tissue composition in bony regions. It is evident that it is close to impossible to control all factors involved, and therefore, there are significant limitations to the current “gold standard.” 9 Immunoassays for biochemical markers of bone resorption were emerging that appear to be sufficiently specific and convenient for clinical use. 10 The need for them arises because the impact of osteoporosis on the aging population increases and better tools to aid in risk prediction and prevention of osteoporosis was mandatory.

Bone resorption markers are important indicators of disease activity in patients with osteoporosis. Normalized results of these indicators are helpful in establishing the disease and its managements. Urinary N-telopeptide has been used for monitoring treatment for osteoporosis for a long time, but now, clinicians are using it to predict the onset of osteoporosis. The new ELISA immunoassay for the urinary excretion of cross-linked collagen peptides is a reliable and specific biochemical marker of bone resorption. 11 The telopeptides are from Type I collagen which forms 90% of organic bone matrix and are cross-linked at N and C terminal ends of the molecules to form the basic fabric and tensile strength of the bone tissue. Urinary N-telopeptide is a sensitive and specific marker of bone resorption. 11 NTx is the stable degradation end product, which can be measured both in serum and urine. The NTx sequence is generated by osteoclastic activity and proteolysis. Hence it does not requires further breakdown or metabolism by kidney or liver for its production. 12 Urinary N-telopeptide value does not significantly vary between male and female, and its range is pretty much same once the patient attains menopause. The urinary excretion is not affected by diet, and therefore shows less variation than the conventional markers. 13

From Table 3 , the independent sample test for urinary N-telopeptide clearly shows significance association with osteoporosis/osteopenia. There is statistical significance of urinary N-telopeptide in study group when compared with the control considering DXA scan as a gold standard. Similarly, urinary N-telopeptide values are significantly higher in the patients who had an associated fracture. The implication from Tables 4 and 5 is that fracture is more common in the patients having osteoporosis/osteopenia. Chi-square test for fracture association in the study group is also statistically significant. Serum calcium, serum phosphorus, and serum alkaline phosphatase did not show significant correlation with urinary N-telopeptide value between study and control groups.

**Table 4:** Study group patients association with fracture

			Groups		Total
			Study	Controls	
Associated with fracture	No	Count	13	44	57
		% Within groups	21.7%	88.0%	51.8%
	Yes	Count	47	6	53
		% Within groups	78.3%	12.0%	48.2%
Total		Count	60	50	110
		% Within groups	100.0%	100.0%	100.0%

**Table 5:** Chi-square test for fracture association with study group

	Value	*df*	Asymptotic significance (two sided)	Exact significance (two sided)	Exact significance (one sided)
Pearson chi-square	48.065	1	0.0005		
Continuity correction	45.445	1	0.000		
Likelihood ratio	52.936	1	0.000		
Fisher's exact test				0.000	0.000
*N* of valid cases	110				

The N-telopeptide is specific to bone due to its unique amino acid sequence. Bone density as measured by DXA provides a static snapshot of bones and does not distinguish if bone loss is ongoing or not. But urinary N-telopeptide is a dynamic measurement of what is actually happening in bone at any given time. There is considerable research ongoing to find a better study to replace the DXA scan. Considering financial parts of the investigations urinary N-telopeptide assessment is being cheaper than DXA. The cost of single region DXA scan is around 2,500 rupees, while urinary N-telopeptide ELISA kit is around 25,000 rupees for 100 patients. If urinary N-telopeptide test were done more frequently, then the kit can be purchased in a much cheaper rate. Moreover, cost of installing DXA, maintaining, and day-to-day running were enormously high compared with a simple urine test such as N-telopeptide. As urinary N-telopeptide is cheap, it can be considered as a screening test also. If we suspect osteoporosis, it is better to go for urinary N-telopeptide and those who test positive can go for current gold standard DXA scan. Thus, combination of these two diagnostic tests could be useful to improve the identification of high risk for fracture.

## Conclusion

Urinary N-telopeptide can give reproducible results and be able to assist in the evaluation of quality of bone and osteoporosis and be a good judge of someone's risk of fracture. Hence, urinary N-telopeptide can be considered as a new diagnostic tool for diagnosing osteoporosis.
